# Healthcare needs and barriers to care among female partners of male suspects of child sexual abuse material offences in Sweden: a qualitative interview study

**DOI:** 10.3389/fpsyt.2025.1618162

**Published:** 2025-10-22

**Authors:** Roberth Adebahr, Josephine Savard, Ulrika Åkerstedt, Markus Byström, Charlotte Sparre, Cecilia Hadding, Katarina Görts Öberg, Jussi Jokinen

**Affiliations:** ^1^ Unit of Psychiatry, Department of Clinical Sciences, Umeå University, Umeå, Sweden; ^2^ ANOVA, Karolinska University Hospital, Stockholm, Sweden; ^3^ Department of Medicine, Karolinska Institutet, Stockholm, Sweden; ^4^ Centre for Psychiatry Research, Department of Clinical Neuroscience, Karolinska Institutet, Stockholm Health Care Services, Region Stockholm, Karolinska University Hospital, Stockholm, Sweden; ^5^ Unit of Professional Development, Department of Clinical Sciences, Umeå University, Umeå, Sweden

**Keywords:** child sexual abuse materials (CSAM), pedophilic disorder, partners and families, barriers accessing health services, sexual offending against children, crisis intervention, mental health service access, counselling

## Abstract

**Objective:**

To explore the impact on female partners of the discovery that their significant other is under investigation for Child Sexual Abuse Material (CSAM) offences, with a focus on the need for professional support.

**Design:**

Semi-structured interviews were conducted and analyzed using reflexive thematic analysis.

**Participants:**

Eight women partnered with men under investigation for CSAM offences.

**Setting:**

ANOVA, a center for sexual medicine, andrology, and trans medicine at Karolinska University Hospital, Stockholm, Sweden.

**Results:**

The findings show severe negative mental health impacts on partners following a disclosure that their significant other had committed CSAM offences. Identified healthcare needs include (1) crisis support in connection with police raid; (2) medical evaluation including suicide risk assessment and follow-up visits; and (3) counseling to manage shame, guilt, self-blame, and ambivalence regarding the future of the relationship. Significant barriers that hinder affected women from seeking and receiving support from friends and family as well as accessing healthcare services were also identified.

**Conclusion:**

Female partners of CSAM offenders have healthcare needs that are currently inadequately met by the Swedish healthcare system. Healthcare professionals, both in primary care and sexual medicine clinics, may significantly contribute to improving treatment for this population. However, there is a need for improvement of healthcare practitioners’ knowledge of the experiences and needs of family members of sexual offenders.

## 1 Introduction

Family members of a sex offender endure adverse social, mental health, and economic consequences solely due to their association with the perpetrator (1–3). This phenomenon is often attributed to the public’s negative perception of sex offenders, which is then projected onto and internalized by their innocent, non-offending family members (4–6). Internet-facilitated offences against children constitute a rapidly escalating worldwide public health issue, with an increasing number of individuals convicted of Child Sexual Abuse Material (CSAM) offences. In Sweden, reports of online CSA to the police rose sharply between 2017 and 2020, more than doubling in just three years. If classified as aggravated, the offence carries a prison sentence of up to six years (7–9).

CSAM offenders have a higher likelihood of cohabitation, marital status, and parenthood, compared to individuals who perpetrate other sexual crimes (10–12). This indicates the likely existence of a substantial cohort of relatives experiencing considerable distress and with healthcare needs that are largely unexplored due to a scarcity of research.

While not explicitly attempting to identify healthcare needs, qualitative studies from Ireland (Kavanagh et al., 2023, 2024), Australia (Liddell et al., 2015; Jones et al., 2022; Salter et al., 2022), United Kingdom (Armitage et al., 2023; Duncan et al., 2022), and USA (Evans et al., 2023) have recently contributed to advancing the understanding of the experiences of relatives of CSAM offenders. Findings highlight important areas for healthcare providers to consider regarding the management and treatment of this group. Firstly, discovering that a loved one is under investigation for CSAM offences is an extremely stressful life event (13–19). Adverse life experiences are the basic hallmark of trauma- and stress-related disorders (e.g., acute stress disorder, adjustment disorder, and post-traumatic stress disorder) and have been associated with the onset of major depressive disorder and a risk of developing or relapsing in substance use disorders (20, 21). Due to the reported major distress among family members of CSAM offenders, several studies highlight the need for crisis support in close connection to the police raid (14–16, 18). Secondly, research indicates an elevated risk of suicide in individuals who have perpetrated sexual crimes against children, particularly those involving CSAM offences (22). Less is known regarding whether this risk is also elevated for their partners, but there are reports of suicidal thoughts and suicide attempts among family members (14, 18). It is therefore essential that the healthcare system establishes protocols to mitigate the risk of suicide. Thirdly, the need to initiate or adjust antidepressant medication has been reported by participants in some studies, representing an additional domain that healthcare services are responsible for evaluating and addressing (14, 18). Lastly, several studies show a lack of mental health support and highlight the need for psychotherapy or counseling to address and manage shame, guilt, grief, and their detrimental effects on self-worth (13–16). Social isolation among partners of sexual offenders is a recurring theme in research. Fear of rumors, anticipated rejection, and experiences of poor treatment frequently prevent relatives from seeking support, both from informal networks and professional services (15). In the Swedish context, social services should serve as an important source of assistance for families with minor children. However, international reports highlight significant shortcomings in children’s services, where the focus is often directed toward risk assessment rather than providing adequate care and support to non-offending family members (23). The current situations for family members of CSAM offenders in a Nordic setting are unknown. Certain cultural and legal features in Nordic countries differ from those in regions where most previous research has been conducted, and these may influence the psychosocial outcomes for partners of CSAM offenders. In the United States, adverse effects on family members have been linked to public sex offender registries, while in the United Kingdom and Ireland, media practices often publicize names and images of offenders, thereby exposing partners to public attention (3, 24). In Sweden, by contrast, there is no public sex offender registry and media are generally restrictive in publishing identifiable information about suspects or victims (25). These contextual features highlight the importance of examining the experiences of partners in a Nordic setting. If there are unmet healthcare needs and barriers to access healthcare services specific to this group, this would hamper the stated goal of the Swedish healthcare system to provide equal access to healthcare as well as ensure good health and treatment on equal terms for all (26).

### 1.1 Objective

To explore the impact on female partners of the discovery that their significant other is under investigation for CSAM offences, with a focus on the need for professional support.

## 2 Method

### 2.1 Study design

This study utilized reflexive thematic analysis (reflexive TA) as described by Braun and Clarke, a method with a flexible and open approach for analyzing qualitative data, suitable for exploratory research with limited prior knowledge about the research questions (27, 28). Reflexive TA allows for comprehensive descriptions of inductively analyzed data from an experiential position, where participants’ experiences and needs can be analyzed at a semantic level, while acknowledging the researcher’s active role in the development, analysis, and reporting of patterns within the data (27–30). As the researcher’s active role is fundamental to reflexive TA, there is a need for transparency about their theoretical assumptions (29). The study was conceptually guided by the principle of equitable, health-promoting healthcare in Sweden, emphasizing accessibility, non-discrimination, prevention, and support irrespective of social or legal status (31). This framework informed the construction of the interview guide, the interview stance, and the analytic process. Within the present study, all authors are healthcare practitioners: RA, MB, CS, and KGÖ are clinical psychologists, UÅ is a psychiatric nurse, and CH, JJ, and JS are board-certified psychiatrists. As such, the researchers’ subjective skills and clinical experience have informed the planning and execution of the present study. The study was conducted at the ANOVA clinic, a center for sexual medicine, andrology, and trans medicine at Karolinska University Hospital, Stockholm, Sweden, where RA, MB, CS, KGÖ, UÅ, and JS work. The primary focus of the sexual medicine unit is treatment of men at risk of committing sexual violence, but support is also frequently provided for their partners and family members.

### 2.2 Participants

The participants (n = 8) were women partnered with men under investigation for CSAM offences. They were between 30 and 63 years old (mean = 46.25, SD = 10.53). All participants had experienced a police raid against their partner. At the time of the police raid, four were married, three were cohabiting, and one was in a living-apart together relationship. In three instances, the participant and the partner had minor children together; in two instances, either the participant or the partner had minor children from a prior relationship; the other couples had either adult children or no children. At the time of the interviews, some women had chosen to end the relationship while others had chosen to stay, although all expressed ambivalence about the future trajectory of their relationship. For simplicity, the term “partner” will be used hereafter for all scenarios. The interval between the police raid and enrollment of the participant in the study ranged from 2 to 7 months (mean = 3.5, SD = 1.66). No partner had received their sentence at the time of the interviews.

### 2.3 Recruitment and data collection

The women were recruited via an ongoing clinical trial at ANOVA that is targeting help-seeking men with a sexual interest in children, in which their partner was participating (32). All subjects in the clinical trial admitted to charges associated with CSAM offences and were clinically assessed by a board-certified psychiatrist as meeting the criteria for DSM-5 pedophilic disorder or hebephilia, i.e., sexual interest in children before puberty or in the first stages of it, respectively (33). If the clinical trial subject gave their consent for their female partner to be contacted, and she agreed to receive a call from research staff, RA or CS reached out to the prospective participant by telephone to offer more information about the qualitative study. A total of ten women were contacted by phone. One was excluded due to insufficient Swedish language skills, and one declined participation. The remaining eight women expressed interest and were invited to ANOVA, where they received written information about the study procedures and provided informed consent. Data were collected via semi-structured interviews, which allowed participants to share their experiences in their own words while ensuring coverage of key areas relevant to the study aim. This approach was considered particularly suitable given the sensitive nature of the topic and the limited prior research. The interview guide was structured around three broad areas: (1) awareness – when and how the participant became aware of their partners sexual interest in children; (2) impact - how this knowledge had affected them personally including emotional, relational, and social consequences; and (3) need for support - their perceived needs and experiences of professional support. Questions were phrased broadly (e.g., impact rather than experienced symptoms, professional rather than healthcare support) to avoid prompting participants to frame their experiences strictly in relation to healthcare services. RA conducted five of the interviews, while CS conducted the remaining three. Prior to the commencement of the interview, participants were given time to review and sign the informed consent form. Participants were thereafter provided with a concise overview of the clinic’s primary aim, the principles of record-keeping, and the objective of the study. Interviews were recorded and the subsequent transcription was outsourced. Initially, ten interviews were planned. However, after the first four interviews had been conducted and transcribed it became apparent that they included exhaustive and complex accounts of experiences, indicating that fewer interviews would suffice to answer the research question (34). The team then decided to cease recruitment after the completion of eight interviews.

### 2.4 Data analysis

The analytic process followed the six phases of reflexive TA, which are: (1) Familiarizing yourself with your data; (2) Generating initial codes; (3) Searching for themes; (4) Reviewing themes; (5) Defining and naming themes; and (6) Producing the report, as described by Braun and Clarke (27, 29). In the first phase, RA listened to all recordings to verify accurate transcription. RA, JS, JJ, and UÅ then read all the interview transcripts several times, while the remaining research team read a subset of the interviews. In the second phase, RA generated initial codes, using NVivo14 (35). RA was also responsible for constructing initial themes and sub-themes in the third phase. In the fourth phase, themes were reviewed by the entire research team; this phase focused on the extent to which the themes and sub-themes represented the coded material and the entire data set. The primary objective of the discussions within the research team was not to attain consensus, but to encourage reflexivity, allowing different medical professionals to act as each other’s “critical friends” offering their specialized expertise and critically examining each other’s perspectives (36). Reviews of themes involved checking for internal homogeneity (e.g., intra-theme coherence) and external heterogeneity (e.g., clear distinctions between themes). During this process, initial themes were collapsed and new sub-themes developed. In this phase, RA re-read and applied all codes to the entire data set. In the next and fifth phase, RA wrote a first draft of the results section, proposed preliminary names for each theme, and selected extracts from the interviews that captured key aspects of the theme. The whole research team then reviewed the results section and collaborated in defining and naming themes and sub-themes to enhance alignment with the essence of each theme/sub-theme and to improve comprehensiveness of the analysis. In the last phase, RA drafted the manuscript, which was then reviewed, edited, improved, and subsequently accepted by all authors.

### 2.5 Quality standards and trustworthiness

Most quality-control methods used in qualitative research, including independent coders and member checking, aim to attain analytical consensus. These techniques are inconsistent with reflexive TA, which emphasizes the researcher’s active role in knowledge production and the reporting of complex results that capture more than one analytic observation (37). This study used the 15-point checklist of criteria for good thematic analysis as outlined by Braun and Clarke (2006) (27). To enhance the trustworthiness of the results, the research group used its broad expertise from different disciplines (medicine, psychology, nursing) and collective clinical experience with the patient group to gain a deeper understanding of the material.

### 2.6 Ethical considerations

The study was approved by The Swedish Ethical Review Authority (ref. no. 2021-02820). It was monitored by an external professional clinical research center, the Karolinska Trial Alliance. Written informed consent was obtained from all participants included in the study. Before the interview, participants were informed about the exceptions to patient confidentiality, including that any violence committed against children can be reported to the police and that healthcare practitioners have an obligation to report any indication that a specific minor or group of minors is at risk of harm to social services as specified in the Swedish Social Services Act. All participants were offered a maximum of three additional psychological consultations after completion of study procedures.

## 3 Results

In analyzing the eight interviews, the authors developed two themes and five sub-themes related to the impact on female partners of the discovery that their significant other is under investigation for CSAM offences, as well as their perceived need of professional support. An overview of themes and subthemes is provided in **Table 1**. The quotes in this section are from specific individuals (P = participant; I = interviewer).

**Table 1:** Themes and sub-themes.

Themes:	Theme one: healthcare needs	Theme two: barriers to care
Sub-themes:	1. Need for crisis support 2. Need for medical evaluation 3. Need for counseling	1. Barriers to seeking support from family and friends 2. Barriers to accessing healthcare

### 3.1 Theme one: healthcare needs

This theme encompasses participants’ descriptions of their various healthcare needs, including their need for crisis support, medical evaluation, and counseling to manage emotional reactions. The identified needs vary over time and require interventions from various healthcare professionals across both primary and specialist care.

#### 3.1.1 Sub-theme 1: need for crisis support

All participants experienced a police raid and in immediate connection to this raid or in the subsequent hours or days, they became aware of their partner’s illegal behavior and sexual interest in children. The police raid, along with the disclosure of their partner’s unlawful online activities and sexual deviance, was consistently described as an extraordinarily stressful experience, both psychologically and emotionally.

*P 5: [crying quietly] It felt like the whole world was falling apart … suddenly you have a husband who has a secret, which is suddenly also my secret … which I don’t even understand, that he has been living with me while doing those things. [sobbing]*

*I: How does that make you feel?*

*P 5: [sobbing] Yes, it’s very difficult, I’ve thought about it a lot. I’m very sad.*

Participants describe their partners as capable, law-abiding persons, and were unaware of their engagement in illegal online activities. “Shock” was the predominant term participants used to describe their initial response to the disclosure.

*P 3: I was completely shocked. I almost fainted and had to lie down on the floor. So, I was shocked, that was my first reaction … and it lasted quite some time.*

All participants reported one or more of the following symptoms: anxiety, sadness, impaired concentration, sleep disturbances, profound fatigue, lack of appetite, and mood swings, often debuting and increasing during a relatively short period of time in relation to disclosure or the police raid.

*P 4: Everyone was sad. I’ve never felt so sad in my life. The first two weeks were a catastrophe; I cried every day, and then … my oldest son had to look after me quite a lot (…) and that is why he has seen his mother so sad, so very sad.*

Shortly following the disclosure, the participants described experiencing exceedingly difficult thoughts and emotions specifically in relation to having a partner who had utilized CSAM. They expressed concern about the safety of their own and other children. Most of the participants also stated that their apprehension extended to the children abused in the material utilized by their partner. One participant recounted that upon encountering her partner for the first time after the disclosure, she immediately started asking questions about potential victims.

*P 2: I begin asking him, and of course, the first question is, “Have you hurt our children?” And he goes, “No! I haven’t; I’ve never touched our children…”. “Have you ever touched another child? Have you hurt your sister’s children or my brother’s children?”. So, I just go through all the kids I can think of.*

Many of the participants described needing immediate crisis support in relation to the police raid. Their own ideas on how such support could be arranged included appointing an individual to accompany law enforcement and safeguard the interests of family members during the raid, the police distributing a business card with a contact number for information and support, or ensuring that family members receive support through social services. Several participants remarked that it might be difficult to actively seek help when in a state of shock.

*P 3: Because you undoubtedly need it [crisis support] during the big shock, but you are incapable of taking that responsibility yourself; someone would have had to force me. Because I was unable to cope, I was worn out, and to seek treatment myself … I would never have been able to take that step on my own.*

#### 3.1.2 Sub-theme 2: need for medical evaluation

Most of the participants interacted with one or more segments of the healthcare system (primary care, occupational health, or psychiatry) or social services, either during a state of acute crisis or in the aftermath of events. Several participants contacted a primary care physician to acquire a prescription for anxiety or sleep medication. In these examples, participants decided not to disclose the real cause of their anxiety symptoms or sleep issues.

*P 4: I called them [primary care] because I needed calming medication, but then I got Atarax [hydroxyzine], I believe it’s called – and then I got Lergigan [promethazine] (…) I only mentioned that something had happened and that I was having panic attacks. I said nothing about the classification [of the offence] I’m not sure if it’s the shame (…) but I don’t want others to feel sorry for me.*

In some cases, participants had received a certificate for medical leave from an employee health services or primary care physician for a couple of weeks to recover. There were also examples of participants who had not been able to complete their studies or had to cut their working hours without seeking healthcare services and requesting medical leave, resulting in negative financial consequences for participants. However, most participants emphasized the importance of continuing to attend work as usual.

*P 5: I sometimes have difficulty focusing or concentrating [sobbing]. And I have not told anyone at work, but I have informed my supervisor that something happened (…), and she has also said, “Do you want to be on medical leave?” And I respond: “No, I don’t want to be at home … it’s better here at work.”*

Some participants described how they developed suicidal ideation during the crisis, which in some cases required inpatient care as a suicide preventative measure.

*P 2: I get to talk to a doctor, and they admit me overnight, and the next day I get to talk to the whole team, and yes, the doctor there looks at me and strains his eyes, and says “No, you’re not sick at all, mentally ill – you’re in total shock and in the middle of a terrible life crisis! And what’s dangerous here is that you might do something desperate when you’re in a panic, therefore I’m going to give you something that will help you calm down”. I asked, “Do you promise?” “Yes, you can stay here as long as you feel it’s necessary”.*

Several participants described how, even though the police raid occurred months prior, they were still far from restored to their pre-crisis state and were struggling with persistent suicidal ideation.

*P 4: But I suppose I also want to run away. I want to leave. I want to quit my job. And I no longer want to be friends with the same people … The point with running away has to do with … that I truly just want to die.*

#### 3.1.3 Sub-theme 3 – need for counseling

The participants described a tendency to prioritize the emotional needs of others over their own need for support. They prioritized the well-being of their children and their partner throughout the immediate crisis, including offering support to other relatives and friends who were shocked to learn what had happened.

*P 2: I’m sitting on the sofa, thinking about what I should do. Should I send him to a hotel, and what should I do? But I thought, “No, he should not be alone!” So, I sent him to our bed [to sleep] next to me and say, “Let’s go to sleep, it’s late,” while I lie awake, making sure he doesn’t go out and commit suicide.*

A basic need was to verbalize thoughts and feelings associated with shame and guilt regarding the partner’s criminal behavior and sexual interests; a further need was support to regulate such feelings.

*P 5: But I’m trying hard not to blame myself [sobbing]. But at the same time, I keep thinking, “Why didn’t I notice anything, why didn’t I see it, was I so naive?” It’s as if I’m struggling against … it’s not me, it’s him.*

Several participants related how feelings of shame triggered thoughts of being unworthy as a romantic partner.

*P 1: (…) And then it was like, “Yes, that’s it, I wasn’t worth loving…”.It may sound pathetic and self-absorbed, but I didn’t believe I was worthy of love; he was with me for the sake of my children, and it was too good to be true…*

These thoughts and feelings were described as having negative effects on self-perception in the long term and participants expressed the need for support to restore their self-worth.

*P 3: I began to feel quite … I lost my confidence. So, why does your husband become attracted to something … well, anyone else is depressing, but it’s considerably worse when it’s a child, especially if you consider yourself a mature woman. Extremely low self-esteem, really.*

All participants expressed ambivalence regarding the decision to remain in their relationship, and the interview situation was often used to search for an answer.

*P 5: I’m not sure how to handle a relationship after something like this – should you keep saying “I forgive you because it’s not your fault that you are the way you are?”… or do you say a firm “No, this is criminal, and I don’t want to be with you anymore?”… that’s also difficult…*

Many stated that understanding the treatment prognosis and the risk of recidivism to unlawful behaviors was essential for them to make judgments about their relationships.

*P 7: Can he receive treatment, or is this an isolated incident that will never happen again? Is this something like “always a pedophile” or…? I need answers to all this.*

Several participants had tried to acquire adequate knowledge about pedophilic attraction and internet-facilitated offences and their link to hands-on crimes. However, obtaining reliable information was difficult and they often lacked someone to talk to about their concerns. Participants’ views on whether to proceed with their relationships was also affected by the reactions of other family members who learned of the partner’s behavior and sexual interest. For example, older children could demand that participants sever ties with their partner.

*P 2: And it’s a very complicated feeling because … well, my children don’t want to have anything to do with my husband; they’ve completely cut off contact. So, this is someone who has been the best father in the world. (…) and that’s precisely how we’ve all felt that my husband was – the pillar of the family, so I can’t simply cut him out of my life. This is not possible.*

This caused conflicts of loyalty within participants’ families, and some expressed a specific need of support to manage strained familial relationships.

In the interviews, participants expressed the need for a safe and nonjudgmental environment in which they could focus on their own reactions and openly talk about their feelings. One participant said that she could only recognize that she possessed agency over her own future within the context of counseling.

*P 7: How I react, how my relationship is, what I end up with … I own that – that realization, this is my mandate, I have full mandate over my own reaction and how I choose to act and how I … eeh, for me it was one of those really … really, really big milestones and it was incredible…*

### 3.2 Theme 2: barriers to care

The participants described a varying degree of need for medical and psychological consultations and treatment, while consistently reporting challenges in obtaining the support that they require. This theme encompasses participants’ descriptions of perceived barriers to care, both to seeking and receiving support from friends and family as well as accessing healthcare services.

#### 3.2.1 Sub-theme 1: barriers to seeking support from family and friends.

Most participants choose to tell someone close to them about their situation, and those who did so described having the support of one or more individuals. However, the decision to share information about the allegations against their partner was often made with careful consideration, weighing the potential risks involved. The taboo nature of the subject was characterized as a barrier that prevented participants from seeking support from those they would typically rely on for support under other circumstances. Participants had concerns about the potential for rumors and the possibility of friends or relatives turning their back on them. All participants described efforts to balance the social risks associated with disclosure against their personal need to talk about their situation; these evaluations entailed complex considerations.

*P 7: Not this weekend, but next weekend, I’m having a friend over … from the place where we used to live … and I know she’ll ask me why we’re getting divorced … And I don’t want to talk about it because she’s such a warm, wonderful person – but she’s not good at keeping her mouth shut. And it won’t be fun … so the only people who know about it are my kids, this friend I came home to immediately after … my parents, and my mother-in-law.*

Participants who chose to remain in the relationship articulated specific concerns regarding whether or not they would be accepted for their decision.

*P 8: But the reason I don’t mention how we live at home or how I avoid it is because I don’t know who will distance themselves and how many [sighing] I understand that there are a lot of people who won’t want to see him anymore, (…) but I’m also concerned about how many people … but I also know that there are those who will do it to me … it’s really difficult.*

Participants who did not share information with people close to them highlighted how they were forced to lie to navigate everyday life; this had a negative impact on their well-being.

*P 6: (…) That’s a significant part of how I feel; it feels like I must lie to everyone, because for example, I must figure out why his brother can’t come home to us [with his children]. And then I must lie…*

Participants with children described having to navigate a new and difficult role of guiding their children regarding the appropriate level of openness about the family situation. Making these decisions was often described as tough, as participants faced situations that were unfamiliar to them while lacking access to others with whom to discuss the decision process. Diverse strategies were employed. For instance, one participant described how she advised her children not to tell anyone due to potential negative consequences.

*P 4: We chose not to tell anyone. And then our eldest son wanted to tell, because he said “I can’t stand this”… and then we said – I’m not sure if it was mean or not, but me and my youngest and my husband … If it comes out … my husband will leave, if his job finds out – he’ll lose his job, of course (…) his friends (…) you don’t know how people will react to this…*

Other participants emphasized the importance of children having autonomy in deciding whether or not they wanted to talk with their peers.

#### 3.2.2 Sub-theme 2: barriers to accessing healthcare services

Despite examples of beneficial experiences with healthcare providers, participants commonly described a reluctance to seek medical attention or conveyed dissatisfaction with treatment and management in the instances when they did seek medical attention. The fact that healthcare providers were required to maintain medical records was seen as an impediment to seek medical attention, since records would then contain information on partners’ behavior and sexual preferences which would be linked to the participant.

*P 5: Yes, when you call someone, especially in healthcare, the first question I ask is, “Do you have confidentiality?” “Yes, but we keep records.” “But what do you write in the records?” (…) It’s extremely difficult. To be always on guard: what do I say and what do I not say? Even if I don’t live with him in ten years, it will still state I did [live with him]. And it’s also for him since he, too, has a life hereafter.*

Participants from smaller towns and rural regions especially expressed that they worried about potential leaks of information from medical records. Furthermore, the obligation of healthcare staff to report any suspected child abuse to social services was regarded as an obstacle to healthcare access. This was especially pronounced for participants who chose to continue their relationship, as they feared that this choice would be viewed as a failure to protect their children and they would thereby be deemed an unfit parent.

*I: Why haven’t you felt able to tell your psychologist [about the allegations against the partner]?*

*P 6: (sobbing) the fear of…. staying with my partner, I’m unable to care for him [her son], for example … so I don’t want a professional, and they call the social services or whatever. My biggest fear is losing him [her son] … [sniffling].*

Additionally, participants described expectations of being met with inadequate knowledge among healthcare professionals about sexual interest in children and internet-related offences, which impeded them from seeking treatment.

*P 8: I’m not sure why I don’t want to go to the primary care center. It doesn’t seem like they … hate to say it, but … are competent enough, if you know what I mean [laughing] in this field; it feels like you want to see someone who has expertise in this topic.*

Inadequate knowledge among healthcare providers was not merely an expectation or presumption, as participants in some cases reported actual experiences of interactions with healthcare providers lacking knowledge.

*P 4: But I believe he was completely taken aback when we told him what the offence was … I believe he told us about you … PrevenTell [The Swedish national telephone help-line for individuals who are concerned about their sexuality] … or whatever it’s called several times, and then I felt as if he almost rejected us, but he was so sweet that it … but he didn’t know much about this. I believe they were more into relationships, like divorces.*

Participants described how healthcare practitioners had to struggle to regulate their own emotions in response to participants’ narratives. Practitioners were reported as failing to maintain their professionalism – such as by expressing shock and dismay at participants’ recounts – a response that participants considered unhelpful.

*P 1: Both professionals and regular folks respond with, “Oh, oh, oh!” “Well, this is something you only read about!” So, what do you do when you don’t get any support?*

Participants also described instances where their symptoms and stressful circumstances were downplayed by healthcare providers, resulting in their exclusion from care despite their desire to initiate contact.

*P 2: I next try to contact primary care, but it does not go well because I am sent to a very difficult nurse who does not understand the gravity of the situation … and she refuses to let me see a doctor.*

## 4 Discussion

This is the first study to focus on healthcare needs and barriers to care among partners of CSAM offenders in a Nordic country. Despite cultural and legal differences compared to the countries where most previous research has been conducted; the findings align with prior studies showing significant adverse impacts on the mental health of family members following the disclosure of a close relative’s offences (13–19). The participants demonstrated a variety of needs including those that require immediate and follow-up interaction with healthcare services such as medical and psychological consultations and treatment, as further discussed below. Additionally, the results support the need for the development of clinical practice guidelines and enhanced accessibility to adequate healthcare services for a population currently experiencing restricted access to healthcare.

All participants express their shock at learning of the allegations against their partners, as they had no previous criminal record and had maintained a typical family life. This narrative is supported by previous research demonstrating that CSAM offenders seldom have a criminal background and commonly maintain a more stable social situation compared to other sexual offenders (12, 38). It is essential for healthcare professionals to acknowledge that these women are often unaware of what is going on inside their own household, and that profound feelings of shame are associated with being misled in this way. Healthcare practitioners must be attentive to their own perspectives to avoid adhering to uninformed narratives that might reinforce these kinds of feelings (39).

As suggested by Duncan et al. (2020), it is vital to provide family members with information, guidance, and support as soon as possible in relation to the police raid; many respondents in this study described a need for – but lack of access to – such support (40). In Sweden, the provision of such support may be restricted due to secrecy regarding the preliminary criminal investigation. However, it might be feasible to provide information, guidance, and support to family members impacted by the offence if the accused consents to share information during the preliminary investigation. A booklet offering information and support to the families of individuals under investigation for CSAM offences has been implemented in the UK; a Swedish equivalent is currently being developed by ANOVA and PrevenTell (41).

Most of the participants experienced symptoms and needs that required the expertise of a physician. Considering the severity of symptoms, physicians should evaluate the suicide risk and need for inpatient care, identify maladaptive coping strategies, assess the need for pharmacotherapy (including anxiolytics and sleep medication), and evaluate the need for medical leave. The results also emphasize the need for follow-up visits to evaluate whether a crisis reaction has progressed into clinical depression or an anxiety condition that may require pharmaceutical intervention (i.e., initiation or modification of antidepressants) and/or psychological treatment. Being a partner of an individual under investigation for CSAM offences does not necessarily lead to the development of a mental health disorder that requires healthcare interventions. Previous research has identified risk factors for developing stress-related disorders after an extreme event; risk factors include sociodemographic factors, personality, pre-exposure psychopathology, as well as genetic and neurobiological factors (42, 43). Partners with these risk factors can be assumed to be at greater risk. However, the considerable adverse effects on mental health seen in the majority of individuals in this study highlights the necessity for further examination of the prevalence of stress and trauma-related disorders, clinical depression, and anxiety disorders in the partners of sexual offenders.

The finding that participants withhold relevant information about what triggered their symptoms when seeking care poses significant risks and may compromise their access to optimal care. While patient concealment of relevant medical information is common, strategies to enhance patients’ openness should be implemented, for instance, by improving trust and communication between patients and their physicians (44, 45). The findings of the present research indicate that participants often seek primary care initially. Increased knowledge among primary healthcare practitioners on sexual deviance, sexual offences, and consequences for offenders’ families could enhance loved one’s openness about their situation and lead to improved accessibility and a sense of confidence in the care that is provided. In 2022, ANOVA launched an online training program for primary care practitioners in Sweden to aid them in assessing and managing individuals with high-risk sexual behaviors. Such initiatives align well with the needs identified in the current study and could serve an important function in lowering barriers to necessary healthcare services, however an empirical evaluation of the effectiveness of the training program is required.

Consistent with prior studies, the present research demonstrates that shame, guilt, and self-blame are key emotional and psychological reactions in the partners of CSAM offenders (14–16, 18, 19). Shame, guilt, and self-blame are interrelated and common responses to extreme events and are linked to the severity of mental health symptoms and suicidality following such events (46, 47). Although specific approaches may differ across various therapeutic practices, an essential element of counseling for partners of CSAM offenders should be the acknowledgement and provision of support to manage these responses (48). In examining the needs of family members, peer support groups often emerge as an appropriate intervention (13). Qualitative research indicates that a group setting, which facilitates the sharing of experiences among individuals in comparable circumstances, may provide emotional and practical support, reducing feelings of shame and isolation while increasing self-confidence and empowerment (24, 49). However, the empirical evidence for the effectiveness of peer support for mental health disorders is inconsistent and the possible adverse effects have received limited research attention (50, 51). For instance, it has been shown that female partners to CSAM offenders who choose to remain in the relationship with the offender found it challenging to participate in peer support initiatives (17, 19). Participants in group psychoeducational therapy led by healthcare professionals can benefit from the peer support of individuals with similar experiences, while also utilizing the expertise of the health practitioners. The extent to which public healthcare can provide support that complements or exceeds what informal networks offer remains an empirical question, and such interventions warrant systematic evaluation.

All participants reported that they were engaged in an ongoing emotional and cognitive process about the future of their relationship. The results indicate that it is essential for participants to explore this ambivalence in a safe setting that provides them with the opportunity to reflect on their own needs and agency. As many respondents expressed concern about being judged for not having left their relationships or having decided to stay in their relationships, an overemphasis by healthcare practitioners on the necessity of ending their relationship risks alienating those seeking care. As such, the current study finds support for the importance of maintaining balanced and nuanced views on the status of the relationship, which aligns with recommendations in Duncan et al. (2020), that women should not be pressured into leaving their partner (40). Some participants expressed that their ambivalence regarding the possibility of maintaining their relationships was associated with a perceived lack of knowledge about recidivism risk, pedophilic attraction, internet-facilitated crimes, and the potential risk for future contact offences. In accordance with Duncan et al. (2020), our results highlight that it is essential for family members to be given accurate, up-to-date information on the risk of reoffending, as well as information about pedophilic disorder and common comorbidities, treatment alternatives, and treatment effectiveness. Providing this sensitive information surpasses the responsibilities of primary care practitioners but would be feasible for healthcare providers specialized in sexual medicine, highlighting the importance of providing pathways for this group to access specialized healthcare services.

Previous studies indicate that partners of both hands-on and internet-facilitated offenders frequently minimize their partner’s crimes, exhibit cognitive distortions regarding the offences, and fail to recognize that the children exploited in CSAM are victims of grave crimes (19, 49, 52). Unlike these previous results, no themes related to minimizing crimes or cognitive distortions regarding offences were explored in the current study, as the data did not provide support for such interpretations. Previous research indicates that female partners of sex offenders who maintain their relationship take on their partner’s cognitive distortions and denial (52). Consistent with this finding, a hypothetical explanation as to why our results differ from previously described results could be that all the participants’ partners had admitted to their criminal activity and a sexual interest in children and therefore did not exhibit as clear cognitive distortions about their actions.

Social support is recognized for its protective role in stressful situations, and healthcare professionals often recommend requesting assistance from loved ones during a crisis reaction (53). Consistent with previous research, the participants are constrained by shame, fear of rumors, and loyalty to partners when seeking help from friends and family (13–16, 18). To prevent offering inadequate advice, it is important that healthcare providers have access to accurate information to comprehend the specific social difficulties encountered by these family members.

The challenge of obtaining support from family and friends could increase the need for healthcare intervention. However, the results indicate that there are significant barriers for participants to seek support from healthcare services. Firstly, participants voice concerns about medical record maintenance. Medical records are crucial for patient safety and must be maintained by healthcare practitioners (54). In Sweden, the electronic health record (EHR) systems are well-developed, enhancing accessibility of medical information across the healthcare system and allowing patients to access their own records via a national patient portal (55). Notwithstanding significant advantages, EHR use raises concerns about the security, privacy, and confidentiality of patient information (56). In accordance with this study’s findings, prior research indicates that patients are more apprehensive about providing information that could affect their family (57). There are, however, possible adaptations in maintaining EHRs in a Swedish setting; access to records is restricted at the ANOVA clinic, rendering them inaccessible to other healthcare providers. Our clinical experience is that individuals seeking help at the clinic find this reassuring. Secondly, participants fear of the involvement of social services and the legal obligation for healthcare practitioners to report suspected child abuse to social services negatively impacted participants’ help-seeking behavior (58). To mitigate barriers to help-seeking behavior, it is essential to provide information concerning the nature of this obligation and explain the role of social services as well as describe the support that may be available to families, children, and adolescents when required. Thirdly, participants described how their presumption that healthcare professionals had insufficient knowledge and understanding of their situation discouraged them from seeking healthcare services. This aligns with previous research where low trust in healthcare practitioners is a known barrier to accessing care and correlates negatively with treatment adherence, particularly among populations experiencing discrimination and prejudice (59).

### 4.1 Strengths and limitations

This study was conducted in a specialist sexual medicine clinic with expertise in supporting partners and family members of individuals at risk of committing or who have committed sexual offences. The participants appeared to perceive the setting as safe and trustworthy, as they openly shared their experiences, thoughts, feelings, and needs. The study’s quality is further strengthened by the research team’s diverse expertise and careful analysis of the material. Potential limitations include the small sample size and that the recruitment pathway may have introduced selection bias. All participants were female long-term partners of male perpetrators who confessed to the crime, sought medical assistance, and requested support for their partners. This limits the transferability of the findings, as relatives in other types of relationships or where the accused denies the offence may have different needs. Also, all interviews were conducted within a maximum of seven months after the arrest, with a mean of 3.5 months. While resilience may develop over time and reduce the need for formal intervention, this early period is often marked by high distress and may represent a critical window for support.

## 5 Conclusion

Our findings show that partners of CSAM offenders experience a wide range of negative mental health effects following a disclosure that their partner has committed CSAM offences and has a sexual interest in children. Furthermore, results showed that there is a lack of sufficient post-disclosure support to manage these negative mental health effects. Healthcare professionals, both those in primary care with knowledge of crisis management and those at specialized sexual medicine clinics with expertise in harmful sexual behaviors, may significantly contribute to improving treatment for this population. However, to provide adequate assessment and support, it is vital for healthcare practitioners to enhance their knowledge about the experiences and needs of family members of sexual offenders.

## Data Availability

The datasets presented in this article are not readily available because of the ethical and logistical concerns related to the sharing of qualitative data. Requests to access the datasets should be directed to roberth.adebahr@umu.se.
